# Supplementary material for: Neurons embedded in loop‐like motifs act as central hubs for brain‐wide integration
Source: J Physiol. 2026 Mar 5;604(7):2958–84. doi: 10.1113/JP289827 (PMC13039282; doi:10.1113/JP289827)
Supplement: Supplementary file 3 — Supplementary Information [file TJP-604-2958-s001.docx]

| **Acronimus** | **Full area name** |
| --- | --- |
| **ACA** | Anterior cingulate area |
| **ACB** | Nucleus accumbens |
| **APN** | Anterior pretectal nucleus |
| **AUD** | Auditory areas |
| **BLA** | Basolateral amygdalar nucleus |
| **BMA** | Basomedial amygdalar nucleus |
| **CA** | Ammon’s horn |
| **CA1** | Field CA1 |
| **CA2** | Field CA2 |
| **CA3** | Field CA3 |
| **CL** | Central lateral nucleus of the thalamus |
| **COA** | Cortical amygdalar area |
| **CP** | Caudoputamen |
| **DG** | Dentate gyrus |
| **DP** | Dorsal peduncular area |
| **EP** | Endopiriform nucleus |
| **EPd** | Endopiriform nucleus, dorsal part |
| **GPe** | Globus pallidus, external segment |
| **IC** | Inferior colliculus |
| **ILA** | Infralimbic area |
| **LD** | Lateral dorsal nucleus of the thalamus |
| **LGd** | Dorsal part of the lateral geniculate complex |
| **LH** | Lateral habenula |
| **LP** | Lateral posterior nucleus of the thalamus |
| **LS** | Lateral septal nucleus |
| **LSc** | Lateral septal nucleus, caudal part |
| **LSr** | Lateral septal nucleus, rostral part |
| **MB** | Midbrain |
| **MD** | Mediodorsal nucleus of the thalamus |
| **MEA** | Medial amygdalar nucleus |
| **MG** | Medial geniculate complex |
| **MOp** | Primary motor area |
| **MOs** | Secondary motor area |
| **MRN** | Midbrain reticular nucleus |
| **MS** | Medial septal nucleus |
| **NB** | Nucleus of the brachium of the inferior colliculus |
| **OLF** | Olfactory areas |
| **ORB** | Orbital area |
| **ORBm** | Orbital area, medial part |
| **OT** | Olfactory tubercle |
| **PAG** | Periaqueductal gray |
| **PIR** | Piriform area |
| **PL** | Prelimbic area |
| **PO** | Posterior complex of the thalamus |
| **POL** | Posterior limiting nucleus of the thalamus |
| **POST** | Postsubiculum |
| **PT** | Parataenial nucleus |
| **RN** | Red nucleus |
| **RSP** | Retrosplenial area |
| **RT** | Reticular nucleus of the thalamus |
| **SCig** | Superior colliculus, motor related, intermediate gray layer |
| **SCm** | Superior colliculus, motor related |
| **SCs** | Superior colliculus, sensory related |
| **SCsg** | Superior colliculus, superficial gray layer |
| **SI** | Substantia innominata |
| **SNr** | Substantia nigra, reticular part |
| **SPF** | Subparafascicular nucleus |
| **SSp** | Primary somatosensory area |
| **SSs** | Supplemental somatosensory area |
| **SUB** | Subiculum |
| **TH** | Thalamus |
| **TT** | Taenia tecta |
| **VAL** | Ventral anterior-lateral complex of the thalamus |
| **VISa** | Anterior area |
| **VISam** | Anteromedial visual area |
| **VISl** | Lateral visual area |
| **VISp** | Primary visual area |
| **VISpm** | Posteromedial visual area |
| **VISrl** | Rostrolateral visual area |
| **VPL** | Ventral posterolateral nucleus of the thalamus |
| **VPM** | Ventral posteromedial nucleus of the thalamus |
| **ZI** | Zona incerta |

**Table S1. Clarification of area acronyms.** The extended name of areas associated with each acronym recorded in the analyzed dataset.


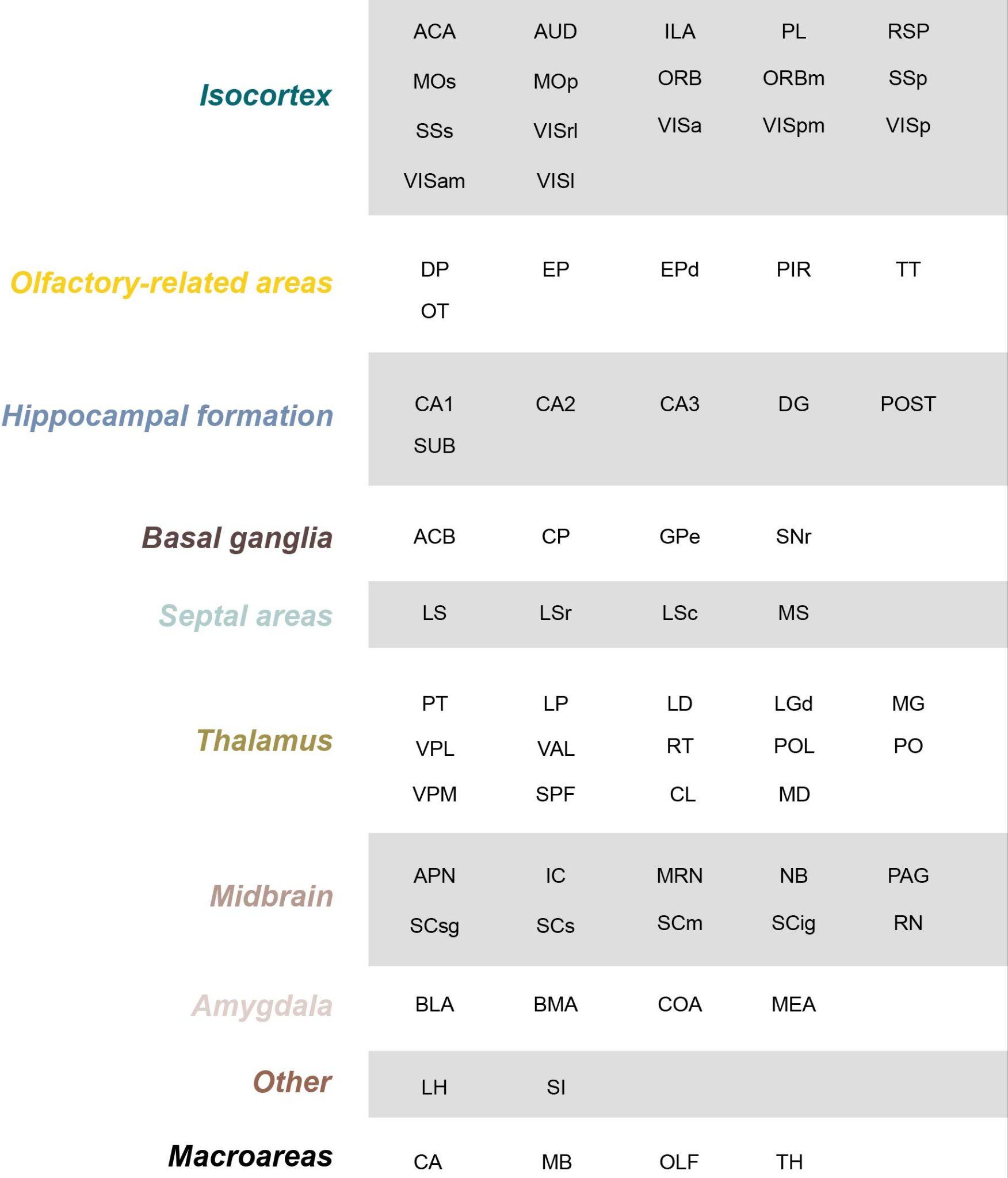


**Figure S1. Sorting of regions within groups.**

Areas included in the analysis were sorted in arbitrary groups indicated in the left column. Names are color-matched with areas depicted in Figure 1.


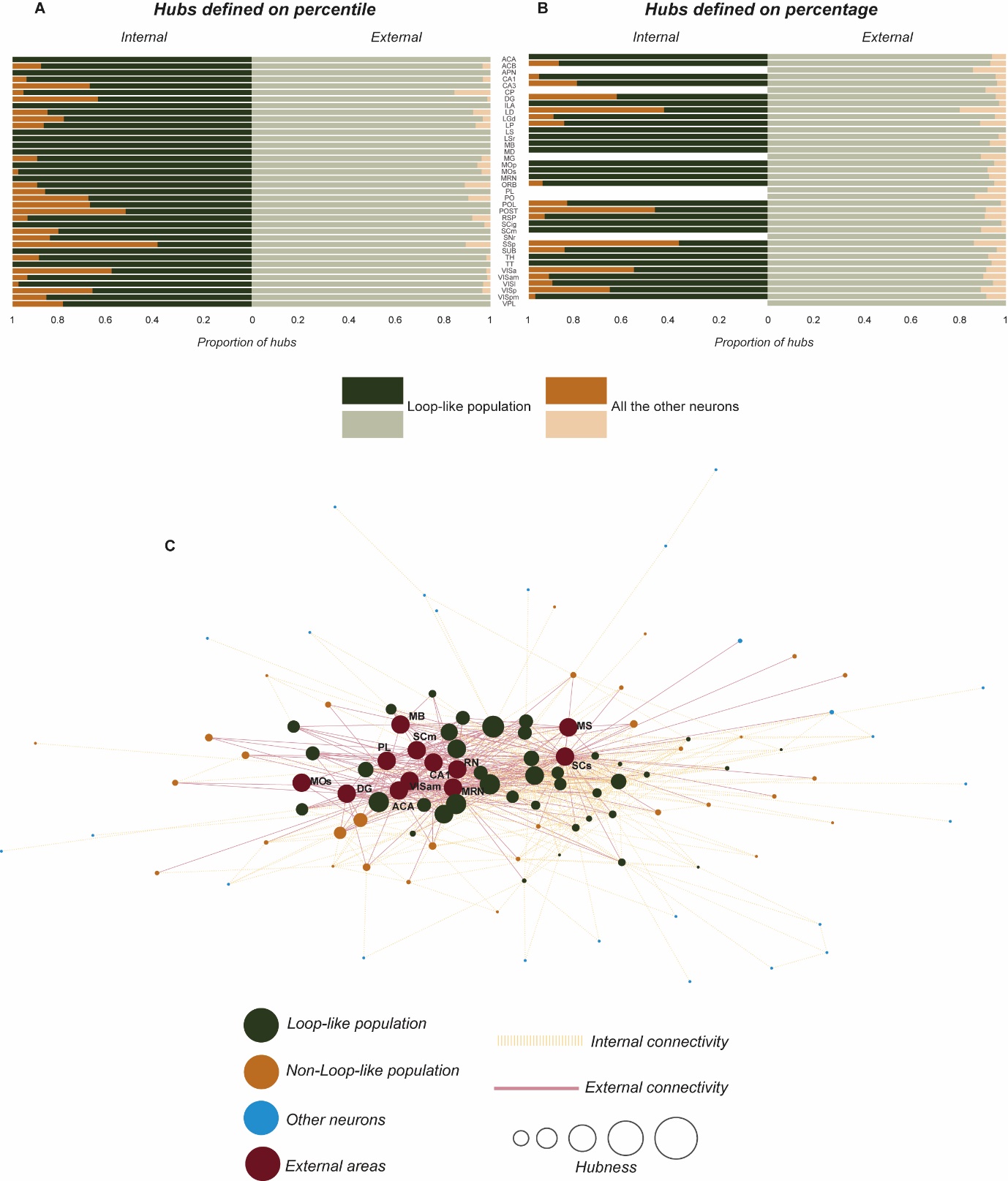


**Figure S2. Relationship between hub neurons and loop-like triplets centered on LGd and comprising all the neurons forming any kind of assembly**

**(A)** Histograms displaying the proportion of internal (left) and external (right) hub neurons, defined as units located in the 95^th^ percentile of the connectivity distribution, in the population forming loop-like (green) or other types of assemblies (orange). **(B)** Same as (**A**) but adopting a definition of “hub neuron” as a unit forming assemblies with more than 50% of external areas it was recorded simultaneously with. **(C)** Graph displaying functional relationships of LGd, in an example session, as edges connecting cells (or entire external areas for red nodes) which form at least one pair assembly. Color and solidness of the edges identify the type of assembly formed, whether intra- or inter-regional. Green and orange nodes identify the population of cells forming loop-like or other types of triplets, while blue nodes represent unlabeled neurons. Node diameter is proportional to hubness attitude, except for red nodes, whose diameter is constant.

**
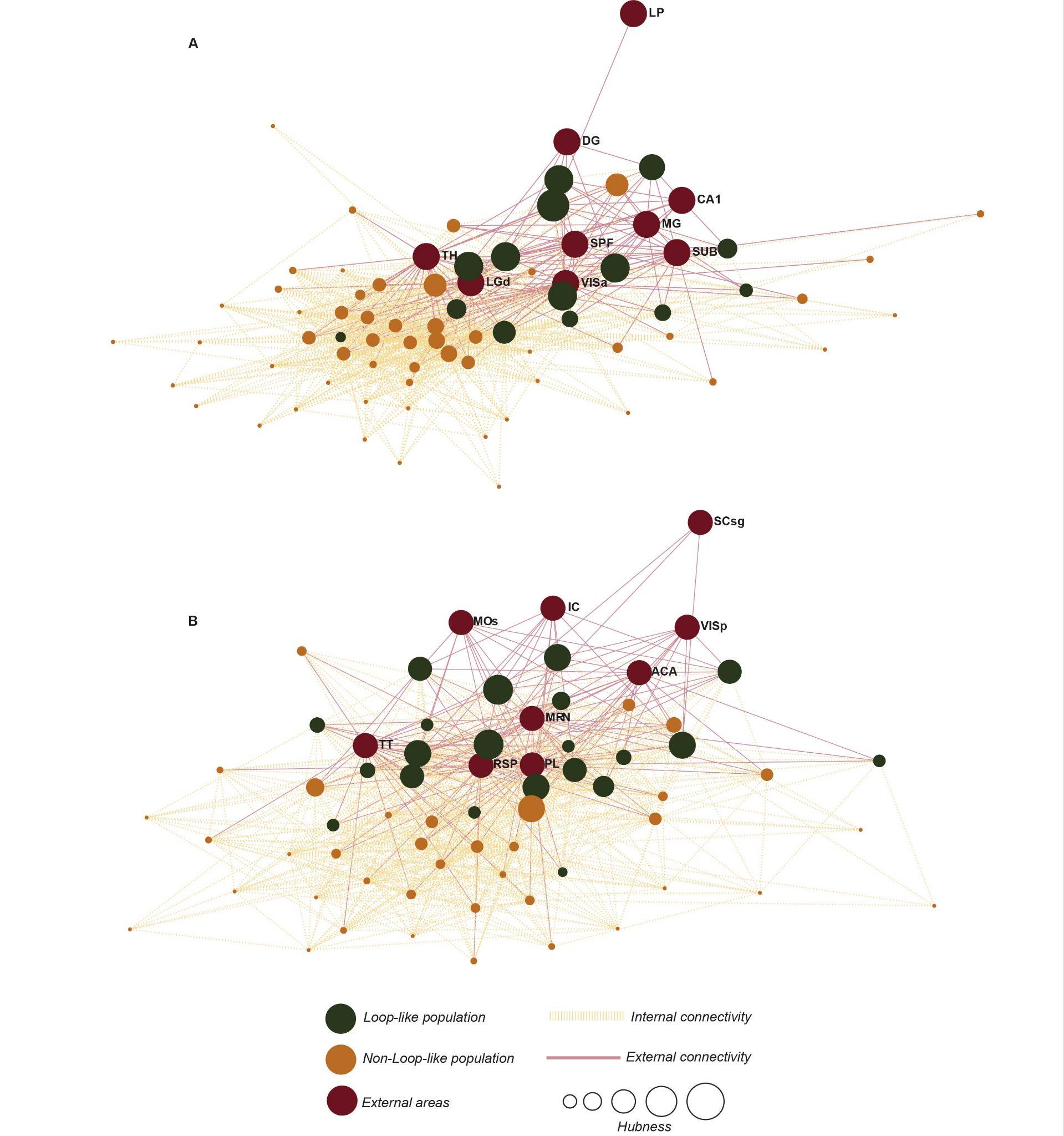
**

**Figure S3. Graph-based representation centered on VISp and ACB**. Graphs centered on **(A)** VISp and **(B)** ACB displaying functional relationships between units belonging to different populations (green, loop-like-forming population; orange, other types of inter-regional triplet-forming population). Solid and dashed edges connect cells to, respectively, external regions (red nodes) or other neurons belonging to their same region, which they form at least one pair assembly with. Except for red nodes, the diameter is proportional to the hubness attitude.
